# Association between very advanced maternal age women with gestational diabetes mellitus and the risks of adverse infant outcomes: a cohort study from the NVSS 2014–2019

**DOI:** 10.1186/s12884-023-05449-0

**Published:** 2023-03-10

**Authors:** Lin Lu, Lidan He, Jifen Hu, Jianhua Li

**Affiliations:** Department of Obstetrics and Gynecology, The First Affiliated Hospital of Fujian Medical University, No.20 Chazhong Road, Taijiang District, Fuzhou, 350005 Fujian China

**Keywords:** Gestational diabetes mellitus, Premature birth, Infant outcomes, Very advanced maternal age, NVSS

## Abstract

**Background:**

To evaluate the association between gestational diabetes mellitus (GDM) and infant outcomes in women of very advanced maternal age (vAMA) (≥45 years).

**Methods:**

This cohort study utilized data from the National Vital Statistics System (NVSS) database (2014–2019) in the United States. Preterm birth was the primary outcome, which was subdivided into extremely preterm, very preterm, and moderate or late preterm. The secondary outcomes were neonatal intensive care unit (NICU) admission, low birthweight and small for gestational age. Univariate and multivariate logistic regression analyses were used to explore the association between GDM and infant outcomes among vAMA women. Subgroup analyses were performed based on race and use of infertility treatment. Odds ratios (ORs) and 95% confidence intervals (CIs) were estimated.

**Results:**

A total of 52,544 vAMA pregnant women were included. All analysis made comparisons between women with vAMA and GDM and women with vAMA and no GDM. Women with GDM had a significantly higher risk of preterm birth than those without GDM (OR = 1.26, 95%CI = 1.18–1.36, *P* < 0.001). Compared with women without GDM, those with GDM had a significantly increased risk of moderate or late preterm birth (OR = 1.27, 95%CI = 1.18–1.37, *P* < 0.001); no significant association of GDM with extremely preterm birth and very preterm birth was observed. Women with GDM had a significantly greater risk of NICU admission than those without (OR = 1.33, 95%CI = 1.23–1.43, *P* < 0.001). GDM was associated with a significantly lower risk of low birthweight (OR = 0.91, 95%CI = 0.84–0.98, *P* = 0.010), and no significant association was found between GDM and small for gestational age (OR = 0.95, 95%CI = 0.87–1.03, *P* = 0.200) in vAMA women.

**Conclusion:**

vAMA women with GDM had an increased risk of preterm birth, especially moderate or late preterm birth. NICU admission and low birthweight were also associated with GDM among vAMA women.

**Supplementary Information:**

The online version contains supplementary material available at 10.1186/s12884-023-05449-0.

## Background

Due to socio-economic development and advances in assisted reproductive technology (ART), the trend of more frequent births among older women, particularly those of very advanced maternal age (≥45 years) (vAMA), is likely to continue [1]. A considerable amount of studies have reported that rising maternal age is one of the key drivers for the increased prevalence of gestational diabetes mellitus (GDM) [2–4]. GDM is traditionally defined as carbohydrate intolerance leading to hyperglycemia of varying severity with onset or first detection during pregnancy [5]. AMA has been identified as a risk factor for GDM [6]. Women of vAMA have a higher incidence of GDM than those under 45 years [7]. Besides, the risks of adverse perinatal outcomes for women aged ≥40 years increase with age [8, 9], and a prior review shows that vAMA women have elevated risks of adverse perinatal outcomes [10]. Extensive research has shown that GDM is associated with increased risks of adverse perinatal outcomes [11–13], such as preterm birth, pre-eclampsia/eclampsia, growth abnormalities, and respiratory distress. Thus, vAMA women who plan to become pregnant may need to pay attention to the risks of GDM and adverse outcomes. Healthcare givers can counsel women of vAMA, especially those with GDM.

Preterm birth (< 37 weeks of gestation) is a common adverse infant outcome, resulting in approximately 1 million infant deaths each year [14]. Even if premature infants survive, they are accompanied by long-lasting diseases that contribute to a global health burden [15, 16]. It has previously been observed that diabetes is a significant risk factor for spontaneous and indicated preterm delivery [11, 17–19]. Diboun et al. [20] indicated that GDM may be used as a novel predictor of preterm delivery. In the study of Billionnet et al. [11], the risk of preterm birth was illustrated to be higher in the GDM group than in the no diabetes group. Regarding other infant outcomes, Venkatesh et al. [21] reported that from 2014 through 2020, the frequency of neonatal intensive care unit (NICU) admission increased, while no significant change was shown in small for gestational age for American women with GDM aged 15–44 years. Although many studies delved into the relationship between GDM and preterm birth, the association of GDM with preterm birth for women of vAMA awaits exploration, which may help identify the population with a high risk of preterm birth and devise prevention and intervention strategies to improve outcomes in vAMA women. The relationships between GDM and NICU admission, low birthweight and small for gestational age are also under-researched.

This study aimed to evaluate the associations of GDM with preterm birth, NICU admission, low birthweight and small for gestational age in vAMA women using the National Vital Statistics System (NVSS) database (2014–2019) in the United States. Given that these associations may vary by race and use of infertility treatment, we further performed subgroup analyses.

## Methods

### Study design and population

This was a cohort study. All data of pregnant women aged 45 or older who were tested for GDM and did not have pre-gestational diabetes were extracted from the NVSS 2014–2019. The NVSS database provides data on births and deaths as well as maternal characteristics in 50 states, New York City, District of Columbia, and 5 territories (Puerto Rico, Virgin Islands, Guam, American Samoa, and Northern Mariana Islands) of the United States [22]. Participants were excluded according to the following criteria: (1) women with infections presenting or treated during this pregnancy; (2) women with missing information on gestational weeks, neonatal weight, and NICU admission records.

### Variables

Preterm birth was the primary outcome of this study, which was defined as births before 37 completed weeks of gestation. The World Health Organization (WHO) further subdivided preterm birth based on gestational age: extremely preterm (< 28 weeks), very preterm (28 to < 32 weeks), and moderate or late preterm (32 to < 37 weeks) [23]. Secondary outcomes were NICU admission, low birthweight and small for gestational age. Low birthweight was defined as a birthweight < 2500 g, and small for gestational age was defined as a birthweight less than the 10th percentile. The following variables were collected: maternal age at delivery (years), race [Asian, Black (Black or African American), White, other (American Indian or Alaska Native, Native Hawaiian or Other Pacific Islander, and more than one race)], education [less than 12 grade, high school/general educational development (GED), some college or associate degree (AA), bachelor or higher], pre-pregnancy weight (lb), pre-pregnancy body mass index (BMI) (BMI < 18.5 kg/m^2^, underweight; BMI = 18.5–24.9 kg/m^2^, normal; BMI = 25.0–29.9 kg/m^2^, overweight; BMI = 30.0–34.9 kg/m^2^, obesity), delivery weight (lb), weight gain (lb), smoking before pregnancy (yes or no), smoking status 1st/2nd/3rd trimester (mother-reported smoking in the three trimesters of pregnancy, yes or no), hypertension eclampsia (yes or no), gestational hypertension (yes or no), pre-pregnancy hypertension (yes or no), number of prenatal visits, the Special Supplemental Nutrition Program for Women, Infants, and Children (WIC, receipt of WIC food for the mother during this pregnancy, yes or no), plurality, prior birth now living, prior birth now dead, prior other terminations, total birth order, gestational age (weeks), newborn sex (female or male), birth weight (g), infertility treatment used (yes or no), pregnancy method (natural pregnancy, pregnancy via ART), method of delivery [spontaneous, non-spontaneous (forceps, vacuum, cesarean)], preterm birth [extremely preterm, very preterm, moderate or late preterm; spontaneous, indicated (forceps, vacuum, cesarean)], NICU admission, low birthweight (yes or no), and small for gestational age (yes or no). WIC is a program intended to help low income pregnant women, infants, and children through age 5 receive proper nutrition by providing vouchers for food, nutrition counseling, health care screenings and referrals; it is administered by the U.S. Department of Agriculture (https://ftp.cdc.gov/pub/Health_Statistics/NCHS/Dataset_Documentation/DVS/natality/UserGuide2019-508.pdf). Infertility treatment referred to using fertility enhancing drugs, artificial insemination, intrauterine insemination, or using ART. ART included in vitro fertilization (IVF), gamete intrafallopian transfer (GIFT), and zygote intrafallopian transfer (ZIFT). Information on variables is available at https://www.cdc.gov/nchs/nvss/index.htm.

### Statistical analysis

Continuous data were tested for normality using the Kolmogorov-Smirnov test, and the continuous data of normal distribution were described as mean ± standard deviation (Mean ± SD), and the t-test was used for comparisons between groups. Non-normally distributed continuous variables were shown by median and quartile [M (Q_1_, Q_3_)], and the Wilcoxon rank sum test was used for comparisons between groups. Categorical data of groups were compared with the Pearson’s χ^2^ test, and expressed as cases and the constituent ratio [n (%)]. Statistical power was calculated (power = 1). Missing data were imputed using multiple imputation (Supplementary Table 1). Data before imputation were also used for multivariate analyses to conduct sensitivity analyses.

In order to study the association between GDM and preterm birth among vAMA women, we established three models, and odds ratios (ORs) with 95% confidence intervals (CIs) were estimated. Model 1 was a univariate model. Model 2 was a multivariate model adjusting for maternal age at delivery, race, education, and newborn sex. Then all variables were included in a multivariable model for stepwise regression, and the following variables were adjusted for in Model 3: maternal age at delivery, race, education, newborn sex, pre-pregnancy weight, pre-pregnancy BMI, delivery weight, weight gain, smoking before pregnancy, hypertension eclampsia, gestational hypertension, pre-pregnancy hypertension, number of prenatal visits, plurality, total birth order, prior birth now living, prior other terminations, birth weight, pregnancy method, and method of delivery. Subgroup analyses were then performed based on race and use of infertility treatment to demonstrate if and how the association between GDM and preterm birth in vAMA women varied by race and use of infertility treatment. Further, preterm birth was subdivided into extremely preterm, very preterm, and moderate or late preterm birth. Logistic regression was used to investigate the association between GDM and different stages of preterm birth. Model 1 was a univariate model. Model 2 was a multivariate model correcting for maternal age at delivery, race, education, and newborn sex. Model 3 was a multivariate model correcting for maternal age at delivery, race, education, newborn sex, delivery weight, smoking status 2nd trimester, hypertension eclampsia, gestational hypertension, pre-pregnancy hypertension, number of prenatal visits, WIC, plurality, prior other terminations, total birth order, birth weight, pregnancy method, and method of delivery. As for the associations of GDM with NICU admission, low birthweight and small for gestational age in vAMA women, analytical methods were the same as those for the association between GDM and preterm birth, and subgroup analyses by race and use of infertility treatment were also conducted for these outcomes.

All statistical analyses were two-sided, and *P* < 0.05 was considered to be statistically significant. All analyses were completed by SAS 9.4 software (SAS Institute Inc., Cary, NC, USA).

## Results

### Participant characteristics

**Fig. 1:**
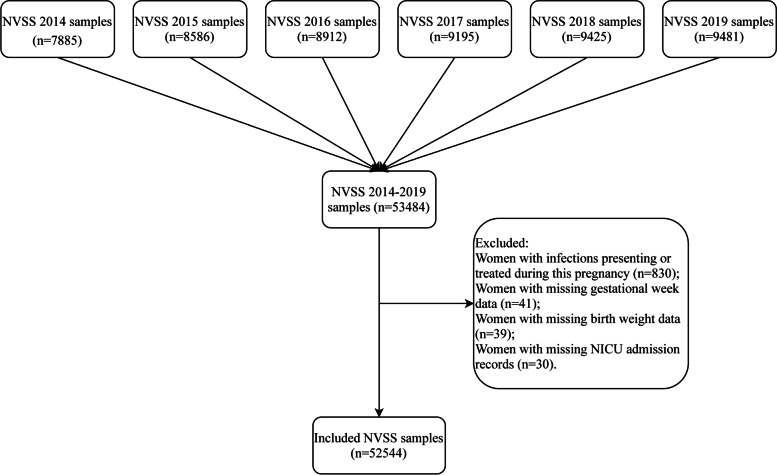
Flow chart of participant selection. NVSS, the National Vital Statistics System; NICU, neonatal intensive care unit

There were a total of 53,484 pregnant women of vAMA in the NVSS database (2014–2019). After excluding women with infections presenting or treated during this pregnancy (*n* = 830), and women with missing information on gestational weeks (*n* = 41), newborn birth weight (*n* = 39), and NICU admission records (*n* = 30), 52,544 pregnant women were included in this study. The follow-up time was 37.62 ± 2.97 weeks. Figure 1 presents the flow chart of participant selection. Among them, 7563 women had GDM, while 44,981 pregnant women did not have GDM. The average age of them was 46.39 ± 1.63 years. The proportion of Asians, Blacks, Whites, and other races was 13.48% (7082), 15.42% (8103), 68.61% (36053), and 2.49% (1306), respectively. The median weight gain during pregnancy was 27.00 (19.00, 36.00) lb. Of the included women, 30.00% (15761) used infertility treatment. Women who had a premature birth accounted for 24.00% (12609) of the total, with 1.31% (689) having an extremely preterm birth, 2.79% (1466) having a very preterm birth, and 19.90% (10454) having a moderate or late preterm birth; 18.10% (9513) of newborns were admitted to the NICU, 18.58% (9761) had a low birthweight, and 9.97% (5241) were small for gestational age. Among preterm birth, the proportions of spontaneous preterm birth and indicated preterm birth were 23.08 and 76.92%, respectively. More details for participant characteristics are shown in Table 1.

**Table 1:** Characteristics of the included population

Variables	Total (***n*** = 52,544)	Pregnant women without GDM (***n*** = 44,981)	Pregnant women with GDM (***n*** = 7563)	Statistics	***P***
Maternal age at delivery, years, Mean ± SD	46.39 ± 1.63	46.40 ± 1.64	46.37 ± 1.62	t = 1.23	0.220
Race, n (%)				χ^2^ = 152.230	< 0.001
Asian	7082 (13.48)	5736 (12.75)	1346 (17.80)		
Black	8103 (15.42)	7050 (15.67)	1053 (13.92)		
White	36,053 (68.61)	31,103 (69.15)	4950 (65.45)		
Other	1306 (2.49)	1092 (2.43)	214 (2.83)		
Education, n (%)				χ^2^ = 375.705	< 0.001
Less than 12 grade	5975 (11.37)	4730 (10.52)	1245 (16.46)		
High school/GED	6920 (13.17)	5738 (12.76)	1182 (15.63)		
Some college or AA	10,203 (19.42)	8631 (19.19)	1572 (20.79)		
Bachelor or higher	29,446 (56.04)	25,882 (57.54)	3564 (47.12)		
Pre-pregnancy weight, lb., Mean ± SD	157.39 ± 35.63	156.01 ± 34.77	165.63 ± 39.36	t = − 19.99	< 0.001
Pre-pregnancy BMI, n (%)				χ^2^ = 914.794	< 0.001
Underweight	926 (1.76)	844 (1.88)	82 (1.08)		
Normal	22,601 (43.01)	20,307 (45.15)	2294 (30.33)		
Overweight	16,180 (30.79)	13,781 (30.64)	2399 (31.72)		
Obesity	12,837 (24.43)	10,049 (22.34)	2788 (36.86)		
Delivery weight, lb., Mean ± SD	185.27 ± 36.18	184.45 ± 35.49	190.15 ± 39.66	t = −11.72	< 0.001
Weight gain, lb., M (Q_1_, Q_3_)	27.00 (19.00, 36.00)	28.00 (20.00, 36.00)	23.00 (15.00, 33.00)	Z = -23.502	< 0.001
Smoking before pregnancy, n (%)				χ^2^ = 2.218	0.136
No	51,401 (97.82)	44,020 (97.86)	7381 (97.59)		
Yes	1143 (2.18)	961 (2.14)	182 (2.41)		
Smoking status 1st trimester, n (%)				χ^2^ = 0.178	0.673
No	51,671 (98.34)	44,238 (98.35)	7433 (98.28)		
Yes	873 (1.66)	743 (1.65)	130 (1.72)		
Smoking status 2nd trimester, n (%)				χ^2^ = 0.001	0.978
No	51,771 (98.53)	44,319 (98.53)	7452 (98.53)		
Yes	773 (1.47)	662 (1.47)	111 (1.47)		
Smoking status 3rd trimester, n (%)				χ^2^ = 0.004	0.947
No	51,805 (98.59)	44,349 (98.59)	7456 (98.59)		
Yes	739 (1.41)	632 (1.41)	107 (1.41)		
Hypertension eclampsia, n (%)				χ^2^ = 5.445	0.020
No	52,234 (99.41)	44,730 (99.44)	7504 (99.22)		
Yes	310 (0.59)	251 (0.56)	59 (0.78)		
Gestational hypertension, n (%)				χ^2^ = 346.242	< 0.001
No	46,470 (88.44)	40,260 (89.50)	6210 (82.11)		
Yes	6074 (11.56)	4721 (10.50)	1353 (17.89)		
Pre-pregnancy hypertension, n (%)				χ^2^ = 320.794	< 0.001
No	49,818 (94.81)	42,967 (95.52)	6851 (90.59)		
Yes	2726 (5.19)	2014 (4.48)	712 (9.41)		
Number of prenatal visits, M (Q_1_, Q_3_)	12.00 (10.00, 14.00)	12.00 (9.00, 14.00)	12.00 (10.00, 15.00)	Z = 11.083	< 0.001
WIC, n (%)				χ^2^ = 279.140	< 0.001
No	41,933 (79.81)	36,437 (81.01)	5496 (72.67)		
Yes	10,611 (20.19)	8544 (18.99)	2067 (27.33)		
Plurality, M (Q_1_, Q_3_)	1.00 (1.00, 1.00)	1.00 (1.00, 1.00)	1.00 (1.00, 1.00)	Z = 0.457	0.648
Prior birth now living, M (Q_1_,Q_3_)	1.00 (0.00, 3.00)	1.00 (0.00, 3.00)	1.00 (0.00, 3.00)	Z = 5.848	< 0.001
Prior birth now dead, Mean ± SD	0.03 ± 0.28	0.03 ± 0.27	0.04 ± 0.30	t = −2.30	0.021
Prior other terminations, M (Q_1_, Q_3_)	0.00 (0.00, 1.00)	0.00 (0.00, 1.00)	0.00 (0.00, 2.00)	Z = 6.863	< 0.001
Total birth order, M (Q_1_, Q_3_)	3.00 (2.00, 5.00)	3.00 (2.00, 5.00)	4.00 (2.00, 5.00)	Z = 8.346	< 0.001
Gestational age, weeks, Mean ± SD	37.62 ± 2.97	37.66 ± 3.00	37.35 ± 2.79	t = 8.75	< 0.001
Newborn sex, n (%)				χ^2^ = 2.426	0.119
Female	25,891 (49.27)	22,227 (49.41)	3664 (48.45)		
Male	26,653 (50.73)	22,754 (50.59)	3899 (51.55)		
Birth weight, g, Mean ± SD	3061.08 ± 709.21	3061.90 ± 710.49	3056.17 ± 701.57	t = 0.65	0.516
Infertility treatment used, n (%)				χ^2^ = 8.487	0.004
No	36,783 (70.00)	31,596 (70.24)	5187 (68.58)		
Yes	15,761 (30.00)	13,385 (29.76)	2376 (31.42)		
Pregnancy method, n (%)				χ^2^ = 9.380	0.002
Natural pregnancy	38,681 (73.62)	33,222 (73.86)	5459 (72.18)		
Pregnancy via ART	13,863 (26.38)	11,759 (26.14)	2104 (27.82)		
Method of delivery, n (%)				χ^2^ = 89.588	< 0.001
Spontaneous	19,594 (37.29)	17,142 (38.11)	2452 (32.42)		
Non-spontaneous	32,950 (62.71)	27,839 (61.89)	5111 (67.58)		
Preterm birth, n (%)				χ^2^ = 59.518	< 0.001
No	39,935 (76.00)	34,452 (76.59)	5483 (72.50)		
Yes	12,609 (24.00)	10,529 (23.41)	2080 (27.50)		
Extremely preterm	689 (1.31)	622 (1.38)	67 (0.89)		
Very preterm	1466 (2.79)	1254 (2.79)	212 (2.80)		
Moderate or late preterm	10,454 (19.90)	8653 (19.24)	1801 (23.81)		
NICU admission, n (%)				χ^2^ = 89.877	< 0.001
No	43,031 (81.90)	37,131 (82.55)	5900 (78.01)		
Yes	9513 (18.10)	7850 (17.45)	1663 (21.99)		
Low birthweight, n (%)				χ^2^ = 4.727	0.030
No	42,783 (81.42)	36,693 (81.57)	6090 (80.52)		
Yes	9761 (18.58)	8288 (18.43)	1473 (19.48)		
Small for gestational age, n (%)				χ^2^ = 1.721	0.190
No	47,303 (90.03)	40,526 (90.10)	6777 (89.61)		
Yes	5241 (9.97)	4455 (9.90)	786 (10.39)		

*GDM* Gestational diabetes mellitus, *SD* Standard deviation, *GED* General educational development, *AA* Associate degree, *BMI* Body mass index, *WIC* the Special Supplemental Nutrition Program for Women, Infants, and Children, *NICU* Neonatal intensive care unit

### Comparisons of characteristics between women with and without GDM

The results illustrated that there were significant differences between pregnant women with and without GDM in race (*P* < 0.001), education (*P* < 0.001), pre-pregnancy weight (*P* < 0.001), pre-pregnancy BMI (*P* < 0.001), delivery weight (*P* < 0.001), weight gain (*P* < 0.001), number of prenatal visits (*P* < 0.001), prior other terminations (*P* < 0.001), total birth order (*P* < 0.001), WIC (*P* < 0.001), gestational age (*P* < 0.001), preterm birth (*P* < 0.001), prior birth now living (*P* < 0.001), prior birth now dead (*P* = 0.021), hypertension eclampsia (*P* = 0.020), gestational hypertension (*P* < 0.001), pre-pregnancy hypertension (*P* < 0.001), NICU admission (*P* < 0.001), method of delivery (*P* < 0.001), low birthweight (*P* = 0.030), infertility treatment used (*P* = 0.004), pregnancy method (*P* = 0.002) (Table 1).

### Association between GDM and preterm birth in vAMA women

**Fig. 2:**
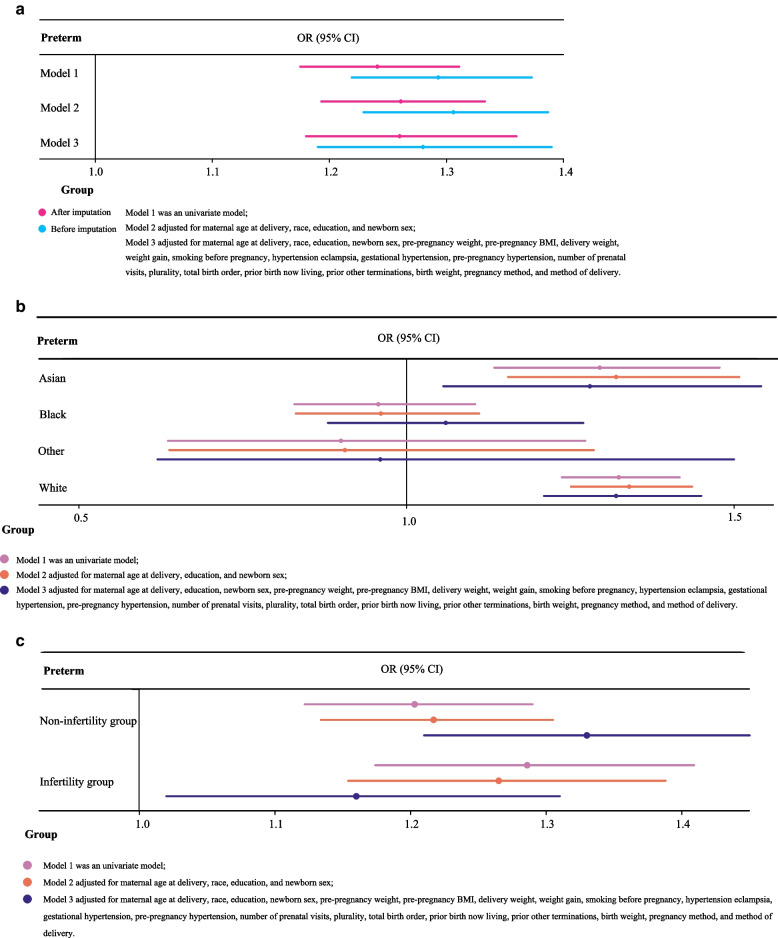
Forest plot for the association between GDM and preterm birth in vAMA women. **a** the association after and before imputation; **b** the association in women of different races; **c** the association in women with and without infertility treatment. GDM, gestational diabetes mellitus; vAMA, very advanced maternal age; OR, odds ratio; CI, confidence interval; BMI, body mass index

Women with GDM had a significantly higher risk of preterm birth than those without GDM, according to multivariate analysis (OR = 1.26, 95%CI = 1.18–1.36, *P* < 0.001). Based on sensitivity analysis, the results were consistent before and after imputation. For different races, it was demonstrated that GDM was associated with a significantly increased risk of preterm birth in Asian (OR = 1.28, 95%CI = 1.08–1.54, *P* = 0.006) and White women (OR = 1.32, 95%CI = 1.21–1.45, *P* < 0.001). GDM was correlated to a significantly greater risk of preterm birth among women without (OR = 1.33, 95%CI = 1.21–1.45, *P* < 0.001) and with (OR = 1.16, 95%CI = 1.02–1.31, *P* = 0.020) infertility treatment (Table 2, Fig. 2a-c).

**Table 2:** Association between GDM and preterm birth in vAMA women

Variables	Model 1^**a**^		Model 2^**b**^		Model 3^**c**^	
	OR (95%CI)	***P***	OR (95%CI)	***P***	OR (95%CI)	***P***
**After imputation**						
GDM						
No	Ref		Ref		Ref	
Yes	1.24 (1.18, 1.31)	< 0.001	1.26 (1.19, 1.33)	< 0.001	1.26 (1.18, 1.36)	< 0.001
Before imputation						
GDM						
No	Ref		Ref		Ref	
Yes	1.29 (1.22, 1.37)	< 0.001	1.31 (1.23, 1.39)	< 0.001	1.28 (1.19, 1.39)	< 0.001
Race						
Asian	1.30 (1.13, 1.48)	0.001	1.32 (1.16, 1.51)	< 0.001	1.28 (1.08, 1.54)	0.006
Black	0.96 (0.83, 1.11)	0.549	0.96 (0.83, 1.11)	0.594	1.06 (0.88, 1.27)	0.545
White	1.32 (1.24, 1.42)	< 0.001	1.34 (1.25, 1.44)	< 0.001	1.32 (1.21, 1.45)	< 0.001
Other	0.90 (0.64, 1.27)	0.551	0.91 (0.64, 1.29)	0.581	0.96 (0.62, 1.50)	0.865
Infertility treatment used						
Non-infertility treatment used	1.20 (1.12, 1.29)	< 0.001	1.22 (1.13, 1.31)	< 0.001	1.33 (1.21, 1.45)	< 0.001
Infertility treatment used	1.29 (1.17, 1.41)	< 0.001	1.27 (1.15, 1.39)	< 0.001	1.16 (1.02, 1.31)	0.020

*GDM* Gestational diabetes mellitus, *vAMA* Very advanced maternal age, *OR* Odds ratio, *CI* Confidence interval, *Ref* Reference, *BMI* Body mass index

For analysis after and before imputation and subgroup analysis by infertility treatment used:

^a^ Model 1 was an univariate model;

^b^ Model 2 adjusted for maternal age at delivery, race, education, and newborn sex;

^c^ Model 3 adjusted for maternal age at delivery, race, education, newborn sex, pre-pregnancy weight, pre-pregnancy BMI, delivery weight, weight gain, smoking before pregnancy, hypertension eclampsia, gestational hypertension, pre-pregnancy hypertension, number of prenatal visits, plurality, total birth order, prior birth now living, prior other terminations, birth weight, pregnancy method, and method of delivery

For subgroup analysis by race:

^a^ Model 1 was an univariate model;

^b^ Model 2 adjusted for maternal age at delivery, education, and newborn sex;

^c^ Model 3 adjusted for maternal age at delivery, education, newborn sex, pre-pregnancy weight, pre-pregnancy BMI, delivery weight, weight gain, smoking before pregnancy, hypertension eclampsia, gestational hypertension, pre-pregnancy hypertension, number of prenatal visits, plurality, total birth order, prior birth now living, prior other terminations, birth weight, pregnancy method, and method of delivery

**Fig. 3:**
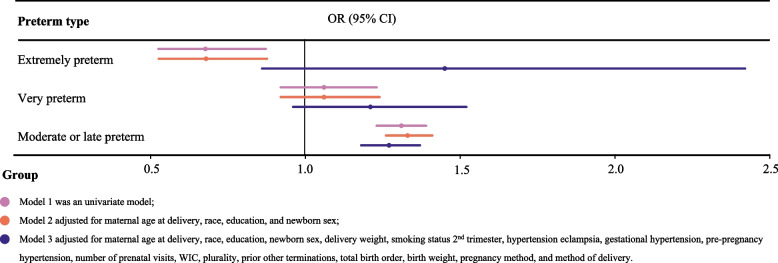
Forest plot for the association between GDM and different stages of preterm birth in vAMA women. GDM, gestational diabetes mellitus; vAMA, very advanced maternal age; OR, odds ratio; CI, confidence interval; WIC, the Special Supplemental Nutrition Program for Women, Infants, and Children

Further, preterm birth was subdivided into extremely preterm birth, very preterm birth, and moderate or late preterm birth. Multivariate analysis showed that compared with women without GDM, those with GDM had a significantly higher risk of moderate or late preterm birth (OR = 1.27, 95%CI = 1.18–1.37, *P* < 0.001); no significant association was observed between GDM and extremely preterm birth and between GDM and very preterm birth (Table 3, Fig. 3).

**Table 3:** Association between GDM and different stages of preterm birth in vAMA women

Preterm birth	Model 1^**a**^		Model 2^**b**^		Model 3^**c**^	
	OR (95%CI)	***P***	OR (95%CI)	***P***	OR (95%CI)	***P***
Extremely preterm	0.68 (0.53–0.87)	0.003	0.68 (0.53–0.88)	0.003	1.45 (0.86–2.42)	0.161
Very preterm	1.06 (0.92–1.23)	0.425	1.06 (0.92–1.24)	0.415	1.21 (0.96–1.52)	0.107
Moderate or late preterm	1.31 (1.23–1.39)	< 0.001	1.33 (1.26–1.41)	< 0.001	1.27 (1.18–1.37)	< 0.001

*GDM* Gestational diabetes mellitus, *vAMA* Very advanced maternal age, *OR* Odds ratio, *CI* Confidence interval, *WIC* the Special Supplemental Nutrition Program for Women, Infants, and Children

^a^ Model 1 was an univariate model;

^b^ Model 2 adjusted for maternal age at delivery, race, education, and newborn sex;

^c^ Model 3 adjusted for maternal age at delivery, race, education, newborn sex, delivery weight, smoking status 2nd trimester, hypertension eclampsia, gestational hypertension, pre-pregnancy hypertension, number of prenatal visits, WIC, plurality, prior other terminations, total birth order, birth weight, pregnancy method, and method of delivery

### Association between GDM and NICU admission in vAMA women

Women with GDM had a significantly greater risk of NICU admission than those without, as illustrated by multivariate analysis (OR = 1.33, 95%CI = 1.23–1.43, *P* < 0.001). Based on sensitivity analysis, the results were consistent before and after imputation. According to subgroup analysis, GDM was associated with a significantly increased risk of NICU admission in Asian (OR = 1.22, 95%CI = 1.01–1.48, *P* = 0.045), Black (OR = 1.35, 95%CI = 1.11–1.63, *P* = 0.003), White (OR = 1.34, 95%CI = 1.22–1.47, *P* < 0.001), and other (OR = 1.65, 95%CI = 1.02–2.66, *P* = 0.040) races; a significant elevated risk of NICU admission was found in women with GDM, regardless of whether they received infertility treatment (OR = 1.31, 95%CI = 1.16–1.49, *P* < 0.001) or not (OR = 1.33, 95%CI = 1.21–1.46, *P* < 0.001) (Table 4).

**Table 4:** Association between GDM and NICU admission in vAMA women

Variables	Model 1^**a**^		Model 2^**b**^		Model 3^**c**^	
	OR (95%CI)	***P***	OR (95%CI)	***P***	OR (95%CI)	***P***
**After imputation**						
GDM						
No	Ref		Ref		Ref	
Yes	1.33 (1.26, 1.42)	< 0.001	1.37 (1.29, 1.46)	< 0.001	1.33 (1.23, 1.43)	< 0.001
**Before imputation**						
GDM						
No	Ref		Ref		Ref	
Yes	1.37 (1.28, 1.46)	< 0.001	1.40 (1.31, 1.50)	< 0.001	1.32 (1.22, 1.44)	< 0.001
**Race**						
Asian	1.35 (1.17,1.57)	< 0.001	1.37 (1.18, 1.59)	< 0.001	1.22 (1.01, 1.48)	0.045
Black	1.15 (0.99, 1.34)	0.070	1.16 (1.00, 1.35)	0.0581	1.35 (1.11, 1.63)	0.003
White	1.40 (1.30, 1.50)	< 0.001	1.434 (1.33, 1.55)	< 0.001	1.34 (1.22, 1.47)	< 0.001
Other	1.23 (0.85, 1.77)	0.282	1.28 (0.88, 1.87)	0.195	1.65 (1.02, 2.66)	0.040
**Infertility treatment used**						
Non-infertility treatment used	1.28 (1.18, 1.38)	< 0.0001	1.31 (1.21, 1.42)	< 0.001	1.33 (1.21, 1.46)	< 0.001
Infertility treatment used	1.40 (1.27, 1.54)	< 0.0001	1.39 (1.26, 1.53)	< 0.001	1.31 (1.16, 1.49)	< 0.001

*GDM* Gestational diabetes mellitus, *NICU* Neonatal intensive care unit, *vAMA* Very advanced maternal age, *OR* Odds ratio, *CI* Confidence interval, *Ref* Reference, *BMI* Body mass index

For analysis after and before imputation and subgroup analysis by infertility treatment used:

^a^ Model 1 was an univariate model;

^b^ Model 2 adjusted for maternal age at delivery, race, education, and newborn sex;

^c^ Model 3 adjusted for maternal age at delivery, race, education, newborn sex, pre-pregnancy weight, pre-pregnancy BMI, delivery weight, weight gain, gestational hypertension, pre-pregnancy hypertension, number of prenatal visits, plurality, total birth order, birth weight, pregnancy method, method of delivery, and preterm birth

For subgroup analysis by race:

^a^ Model 1 was an univariate model;

^b^ Model 2 adjusted for maternal age at delivery, education, and newborn sex;

^c^ Model 3 adjusted for maternal age at delivery, education, newborn sex, pre-pregnancy weight, pre-pregnancy BMI, delivery weight, weight gain, gestational hypertension, pre-pregnancy hypertension, number of prenatal visits, plurality, total birth order, birth weight, pregnancy method, method of delivery, and preterm birth

### Association between GDM and low birthweight in vAMA women

Multivariate analysis demonstrated that in contrast to women without GDM, those with GDM had a significantly decreased risk of low birthweight (OR = 0.91, 95%CI = 0.84–0.98, *P* = 0.010). Based on sensitivity analysis, the results were consistent before and after imputation. GDM was related to a significantly lower risk of low birthweight among Blacks (OR = 0.80, 95%CI = 0.66–0.96, *P* = 0.019). In women without infertility treatment, GDM was associated with a significantly reduced risk of low birthweight (OR = 0.87, 95%CI = 0.79–0.96, *P* = 0.006) (Table 5).

**Table 5:** Association between GDM and low birthweight in vAMA women

Variables	Model 1^**a**^		Model 2^**b**^		Model 3^**c**^	
	OR (95%CI)	***P***	OR (95%CI)	***P***	OR (95%CI)	***P***
**After imputation**						
GDM						
No	Ref		Ref		Ref	
Yes	1.07 (1.01, 1.14)	0.030	1.10 (1.03, 1.17)	0.004	0.91 (0.84, 0.98)	0.010
Before imputation						
GDM						
No	Ref		Ref		Ref	
Yes	1.10 (1.03, 1.18)	0.005	1.12 (1.05, 1.20)	0.001	0.91 (0.84, 0.98)	0.020
Race						
Asian	1.24 (1.08, 1.43)	0.003	1.28 (1.11, 1.48)	0.001	0.95 (0.80, 1.13)	0.571
Black	0.85 (0.73, 0.99)	0.042	0.87 (0.74, 1.02)	0.083	0.80 (0.66, 0.96)	0.019
White	1.09 (1.01, 1.18)	0.024	1.12 (1.03, 1.21)	0.005	0.91 (0.83, 1.00)	0.051
Other	0.75 (0.50, 1.13)	0.162	0.81 (0.54, 1.24)	0.333	0.68 (0.40, 1.17)	0.163
Infertility treatment used						
Non-infertility treatment used	0.94 (0.86, 1.02)	0.112	0.97 (0.89, 1.05)	0.43	0.87 (0.79, 0.96)	0.006
Infertility treatment used	1.27 (1.15, 1.40)	< 0.001	1.24 (1.13, 1.37)	< 0.001	0.96 (0.85, 1.09)	0.551

*GDM* Gestational diabetes mellitus, *vAMA* Very advanced maternal age, *OR* Odds ratio, *CI* Confidence interval, *Ref* Reference, *BMI* Body mass index

For analysis after and before imputation and subgroup analysis by infertility treatment used:

^a^ Model 1 was an univariate model;

^b^ Model 2 adjusted for maternal age at delivery, race, education, and newborn sex;

^c^ Model 3 adjusted for maternal age at delivery, race, education, newborn sex, pre-pregnancy weight, pre-pregnancy BMI, delivery weight, weight gain, smoking before pregnancy, smoking status 1st trimester, hypertension eclampsia, gestational hypertension, pre-pregnancy hypertension, number of prenatal visits, plurality, prior birth now living, prior other terminations, pregnancy method, and method of delivery

For subgroup analysis by race:

^a^ Model 1 was an univariate model;

^b^ Model 2 adjusted for maternal age at delivery, education, and newborn sex;

^c^ Model 3 adjusted for maternal age at delivery, education, newborn sex, pre-pregnancy weight, pre-pregnancy BMI, delivery weight, weight gain, smoking before pregnancy, smoking status 1st trimester, hypertension eclampsia, gestational hypertension, pre-pregnancy hypertension, number of prenatal visits, plurality, prior birth now living, prior other terminations, pregnancy method, and method of delivery

### Association between GDM and small for gestational age in vAMA women

No significant association was found between GDM and a risk of small for gestational age, as exhibited by multivariate analysis (OR = 0.95, 95%CI = 0.87–1.03, *P* = 0.200). Based on sensitivity analysis, the results were consistent before and after imputation. As regards different races, White women with GDM had a significantly decreased risk of small for gestational age (OR = 0.89, 95%CI = 0.80–0.99, *P* = 0.043). There was no significant association between GDM and small for gestational age in women with non-infertility treatment and infertility treatment (Table 6).

**Table 6:** Association between GDM and small for gestational age in vAMA women

Variables	Model 1^**a**^		Model 2^**b**^		Model 3^**c**^	
	OR (95%CI)	***P***	OR (95%CI)	***P***	OR (95%CI)	***P***
**After imputation**						
GDM						
No	Ref		Ref		Ref	
Yes	1.06 (0.97, 1.14)	0.190	1.07 (0.98, 1.16)	0.126	0.95 (0.87, 1.03)	0.200
**Before imputation**						
GDM						
No	Ref		Ref		Ref	
Yes	1.04 (0.95, 1.13)	0.402	1.05 (0.96, 1.14)	0.324	0.92 (0.84, 1.01)	0.068
**Race**						
Asian	1.27 (1.07, 1.52)	0.007	1.29 (1.08, 1.55)	0.004	1.08 (0.89 1.31)	0.438
Black	0.92 (0.75, 1.12)	0.393	0.93 (0.76, 1.14)	0.495	0.89 (0.72, 1.09)	0.260
White	1.01 (0.91, 1.12)	0.825	1.02 (0.92, 1.14)	0.687	0.89 (0.80, 0.99)	0.043
Other	1.26 (0.79, 2.00)	0.334	1.35 (0.84, 2.15)	0.212	1.29 (0.78, 2.16)	0.324
**Infertility treatment used**						
Non-infertility treatment used	0.96 (0.87, 1.07)	0.453	0.98 (0.88, 1.08)	0.655	0.91 (0.81, 1.01)	0.076
Infertility treatment used	1.21 (1.07, 1.38)	0.003	1.19 (1.05, 1.36)	0.007	1.03 (0.90, 1.18)	0.705

*GDM* Gestational diabetes mellitus, *vAMA* Very advanced maternal age, *OR* Odds ratio, *CI* Confidence interval, *Ref* Reference

For analysis after and before imputation and subgroup analysis by infertility treatment used:

^a^ Model 1 was an univariate model;

^b^ Model 2 adjusted for maternal age at delivery, race, education, and newborn sex;

^c^ Model 3 adjusted for maternal age at delivery, race, education, newborn sex, pre-pregnancy weight, weight gain, smoking status 1st trimester, hypertension eclampsia, gestational hypertension, pre-pregnancy hypertension, plurality, prior birth now living, pregnancy method, and method of delivery

For subgroup analysis by race:

^a^ Model 1 was an univariate model;

^b^ Model 2 adjusted for maternal age at delivery, education, and newborn sex;

^c^ Model 3 adjusted for maternal age at delivery, education, newborn sex, pre-pregnancy weight, weight gain, smoking status 1st trimester, hypertension eclampsia, gestational hypertension, pre-pregnancy hypertension, plurality, prior birth now living, pregnancy method, and method of delivery

## Discussion

The present study assessed the association between GDM and adverse infant outcomes (preterm birth, NICU admission, low birthweight and small for gestational age) in vAMA women applying data from the NVSS database. GDM was identified to be positively associated with the risk of preterm birth, especially moderate or late preterm birth; the risks of NICU admission and low birthweight were correlated with GDM in women of very advanced age. According to a prior meta-analysis, women ≥35 years old were more likely to have GDM and worse perinatal outcomes including preterm delivery, low birthweight infants and higher rates of NICU admission [24], which was also supported by Frick et al. [25] and Carolan et al. [26] Fuchs et al. found that women aged 40 years and over had a greater risk of preterm birth [27]. The risk of small-for-gestational-age infants was approximately doubled in vAMA women compared with women aged 35–39 years [28]. As for the relationship between GDM and infant outcomes, Billionnet et al. [11] reported the elevated risks of preterm birth and macrosomia in women with an average age of 30 years having GDM versus those having no diabetes. A cohort study of 46,230 deliveries found that GDM was correlated with a significantly higher risk of spontaneous preterm birth [29]. GDM was associated with mild increases in birth size, as shown by other authors [30]. Few previous studies have focused on the association between GDM and infant outcomes among vAMA women. This study filled this gap, and identified vAMA women with higher risks of infant outcomes. Measures targeting GDM may be adopted to manage these risks for women of vAMA.

As for the possible causes of the association between GDM and preterm delivery in vAMA women, GDM has been associated with polyhydramnios [31, 32], and polyhydramnios can lead to increased uterine tension, and thus induce uterine contractions and cause premature birth. As shown by Buen et al., polyhydramnios acts as a risk factors for preterm delivery [33]. The relatively poor intrauterine environment of vAMA women, which is not conducive to the growth and development of the fetus, may contribute to the positive association of GDM and preterm birth. Additionally, consistent declines in β cell function and insulin secretion are symbols of aging in humans [34–37], and aging effects interact with diabetes to accelerate the progression of many common diabetes complications [38], which may make the association of GDM with premature birth more significant among vAMA women. Considering the finding that over 75% of preterm births were indicated preterm deliveries, clinical practice patterns may play an important role. Indicated delivery is usually chosen for preterm birth in women of very advanced age in clinical practice. Since the physical strength, productivity, cervical elasticity and dilatation ability of vAMA women are inferior to those of young people, indicated preterm delivery (forceps, vacuum, cesarean) may reduce the risk of adverse pregnancy outcomes and complications among these women. Of note, we further showed that the above relationship existed between GDM and moderate or late preterm birth. In clinical practice, more attention should be paid to vAMA women with GDM, and appropriate measures can be taken to reduce risks. The correlations of GDM with NICU admission and low birthweight in vAMA women were also revealed in the current study. More investigations are warranted to consolidate our findings.

Interestingly, we found that the association between GDM and preterm birth varied by race in vAMA women. GDM was associated with a significantly higher risk of preterm delivery among Asians and Whites, while no association was identified among Blacks and other races. Some studies pointed out that in the United States, non-Hispanic Black race (compared with non-Hispanic White race) was a risk factor for preterm birth [39, 40], which did not seem to cohere with our results. However, increased age may have an important influence on the relationship of GDM and preterm birth in different races, which these studies did not take into consideration. Thus, it is worth paying more attention to the effect of vAMA on this relationship. The relationship between GDM and low birthweight was shown to vary by race and use of infertility treatment, and White women with GDM were at a significantly reduced risk of having small-for-gestational-age infants. Further studies are required for validation, and corresponding management of infant outcomes can be undertaken for populations with different risks.

Our study has several strengths. A large, nationally representative sample size (*n* = 52,544) with adequate power (power = 1) was utilized to assess the associations between GDM and adverse infant outcomes (preterm birth, NICU admission, low birthweight and small for gestational age) in pregnant women of very advanced age, making the results reliable. Different stages of preterm birth were also analyzed, and the aforementioned associations were further evaluated according to race and use of infertility treatment, which provides additional insights into these associations for different populations.

A few limitations of the present study need to be noted. First, this study was retrospective in nature, and some data were missing during data collection. To address this, missing data were imputed using multiple imputation, and sensitivity analyses confirmed the reliability of the results. Additionally, the level of evidence for this study is low. Prospective studies are needed for verification. Second, there are no data on some covariates, such as caesarean (elective and emergency), physical activity, psychological and social stress and depression during pregnancy, blood glucose, medication use (especially psychotropic medication), and socioeconomic factors (apart from education and WIC), which may influence our results. Third, this study focused on the U.S. population and has limited generalizability. Future studies are required to investigate the relationship between GDM and infant outcomes in vAMA women with consideration of the above covariates, so as to confirm our findings. This relationship can also be evaluated in populations from other countries. Notably, 76.92% of preterm births were caused by indicated delivery. Hypertension and the pregnancy method may be related to indicated preterm delivery in vAMA women. Future research can investigate the indications for indicated preterm delivery, and assess whether hypertension and the pregnancy method are associated with indicated preterm delivery.

Based on our findings, vAMA women with GDM had higher risks of preterm birth and NICU admission than those without. Greater attention should be paid to vAMA women with GDM and early interventions should be taken to lower the risks. Improving GDM may be a viable approach. Since populations grouped by age and use of infertility treatment had different risks of adverse infant outcomes, individualized measures should be developed. vAMA women with pregnancy planning should be informed of increased risks of preterm birth and NICU admission when they had GDM and corresponding advice can be provided by clinicians or healthcare givers.

## Conclusion

GDM was associated with an increased risk of preterm birth, especially moderate or late preterm birth; the risks of NICU admission and low birthweight were correlated with GDM among vAMA women. More investigations are warranted to verify this conclusion.

## Supplementary Information

**Additional file 1: Supplementary Table 1.** Proportion of missing values.

## Data Availability

The datasets generated and/or analyzed during the current study are available in the NVSS repository, https://www.cdc.gov/nchs/nvss/index.htm.

## Abbreviations

AA: Associate degree
BMI: Body mass index
CIs: Confidence intervals
GDM: Gestational diabetes mellitus
GED: General educational development
Mean ± SD: Mean ± standard deviation
M (Q_1_, Q_3_): Median and quartile
NICU: Neonatal intensive care unit
NVSS: National Vital Statistics System
ORs: Odds ratios
vAMA: Very advanced maternal age
WIC: the Special Supplemental Nutrition Program for Women, Infants, and Children
