# Supplementary material for: Association between very advanced maternal age women with gestational diabetes mellitus and the risks of adverse infant outcomes: a cohort study from the NVSS 2014–2019
Source: BMC Pregnancy Childbirth. 2023 Mar 10;23:158. doi: 10.1186/s12884-023-05449-0 (PMC9999489; doi:10.1186/s12884-023-05449-0)
Supplement: Supplementary file 1 — Additional file 1: Supplementary Table 1. Proportion of missing values. [file 12884_2023_5449_MOESM1_ESM.docx]

**Supplementary table 1.** Proportion of missing values

| **Variables** | **Missing values, n (%)** |
| --- | --- |
| Education | 3434 (6.54%) |
| Pre-pregnancy weight | 1825 (3.47%) |
| Pre-pregnancy BMI | 2054 (3.91%) |
| Delivery weight | 1209 (2.30%) |
| Weight gain | 2458 (4.68%) |
| Smoking cigarettes before pregnancy | 406 (0.77%) |
| Smoking status 1^st^ trimester | 411 (0.78%) |
| Smoking status 2^rd^ trimester | 412 (0.78%) |
| Smoking status 3^rd^ trimester | 557 (1.06%) |
| Number of prenatal visits | 1620 (3.08%) |
| WIC | 984 (1.87%) |
| Prior birth now living | 223 (0.42%) |
| Prior birth now dead | 268 (0.51%) |
| Prior other terminations | 370 (0.70%) |
| Total birth order | 444 (0.85%) |
| Method of delivery | 20 (0.04%) |

BMI, body mass index; WIC, the Special Supplemental Nutrition Program for Women, Infants, and Children.
